# Atypical Psuedo-Demons-Meigs Syndrome Presenting As Acute Dyspnoea With Pseudomembranous Colitis

**DOI:** 10.7759/cureus.52689

**Published:** 2024-01-21

**Authors:** Sanjeev G Gianchandani Gyani, Meenakshi Yeola, Resha O Keshwani, Sachin G Gianchandani, Pankaj Katariya

**Affiliations:** 1 Surgery, Jawaharlal Nehru Medical College, Datta Meghe Institute of Higher Education and Research, Wardha, IND; 2 Minimal Access and Robotic Surgery, Anglia Ruskin University, Chelmsford, ARE; 3 General Surgery, All India Institute of Medical Sciences, Mangalgiri, Mangalgiri, IND; 4 Pediatrics, Jawaharlal Nehru Medical College, Datta Meghe Institute of Higher Education and Research, Wardha, IND; 5 Physiotherapy, Ravi Nair Physiotherapy College, Datta Meghe Institute of Higher Education and Research, Wardha, IND; 6 General Surgery, Jawaharlal Nehru Medical College, Datta Meghe Institute of Higher Education and Research, Wardha, IND

**Keywords:** atypical pseudo-meigs syndrome, ascites, hydrothorax, granulosa theca cell tumour, ovarian mass, demons-meigs syndrome, meigs syndrome

## Abstract

Demons-Meigs syndrome is a rare clinical presentation of benign ovarian mass with hydrothorax and ascites. As ascites can be present in any ovarian mass, hydrothorax is a salient feature of the syndrome. The syndrome is subtyped as atypical in the absence of ascites from the triad. Nevertheless, it is labeled as pseudo-Demons-Meigs syndrome if the ovarian tumor is neoplastic rather than benign. The management of Demons-Meigs syndrome is complex and could be misleading due to pleural effusion and ascites, so an understanding of the syndrome is important. This case report is unique as it has two rare findings of neoplastic tumor and absence of ascites. Furthermore, this case is distinct as both ovaries are involved in malignant granulosa theca cell tumor with right-sided pleural effusion without ascites.

## Introduction

The syndrome described by Demons and Meigs is a rare clinical entity associated with ovarian tumors [1,2]. Demons-Meigs syndrome is a triad of ovarian tumor, ascites, and hydrothorax. Furthermore, it is essential to understand the syndrome to avoid any dilemmas in treating the patient. The patients with female genital tract tumors with ascites and hydrothorax were considered "hopeless" until Demons advised a paradigm shift in the treatment [3]. Demons was the first to suggest not to delay the surgery in the wait to cure hydrothorax [4,5]; rather, excise the ovarian mass after stabilization of the patient. Spiegelberg O (1866), Thomas G (1877), Cillingworth C (1879), Wells S (1882), Demons AJO (1887, 1902, and 1903), and Tait RL (1892) described the syndrome in different modus operandi in literature [6-14].

Culligworth was the first to report fibroma as a cause of Demons-Meigs syndrome in the patient's autopsy, and Demons was the first to suggest excision of the tumor to cure the disease [11-13]. Moreover, Meigs reviewed 124 cases from the literature and described the syndrome in detail leading to a better understanding and awareness of the condition [15]. Furthermore, Rhoads and Terrell were the first to term the condition as Meigs syndrome, and Meigs JV mentioned the term Demons-Meigs syndrome acknowledging the work of Demons [16]. Meigs JV [17-21] defined three characteristics of the syndrome in The American College of Obstetricians and Gynecologists in May 1945. Moreover, atypical or incomplete Demons-Meigs syndrome has pleural effusion and a benign ovarian tumor without ascites [22]. Tjamal's syndrome has features of Meigs syndrome without an ovarian tumor. It is characterized by elevated cancer antigen 125 (CA 125) levels in an individual with systemic lupus erythematosus and other features of Meigs syndrome, including the presence of ascites and hydrothorax. It is called pseudo-pseudo-Meigs syndrome (pseudo is written twice). Due to the absence of ovarian mass, it could be considered a fallacy of pseudo-Meigs syndrome [23-25]. Tjamal's syndrome is also labeled in patients with other connective tissue disorders, which is rare [26]. Demons-Meigs syndrome is an infrequent phenomenon creating confusion primarily due to pleural effusion and ascites. This can mislead the treatment, so it is paramount to understand the syndrome well for appropriate patient management.

## Case presentation

**Figure 1:**
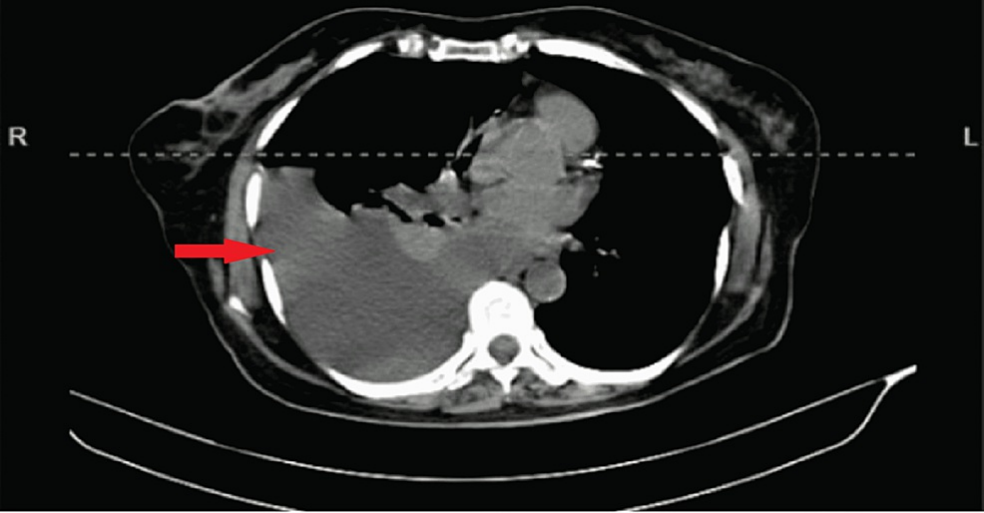
CT scan revealed a massive pleural effusion on the right side CT: Computed tomography

A 67-year-old female reported to the emergency department with severe breathlessness. On evaluation, she had tachypnea, tachycardia, and oxygen saturation of 90% on room air. She had diminished air entry on the right side of the chest with a dull percussion note. X-ray chest revealed a right-sided massive pleural effusion. An immediate needle thoracocentesis was performed, draining 1100 ml of fluid, and the patient was shifted to the ICU and stabilized. However, she had a recurrence of right pleural effusion 40 hours post-initial needle thoracocentesis, which was confirmed on a computed tomography (CT) scan (Figure 1) for which an inter-costal drain was inserted. On detailed history, the patient had noticed an asymptomatic lump in the abdomen 10 months back.

Furthermore, the patient complained of increased frequency of micturition and loose stools for the last nine months. The patient also complained of occasional abdominal cramps and occasional fever. The patient had consulted general practitioners and had received medications multiple times in the last six months, including ciprofloxacin, metronidazole, pantoprazole, and pain relief. She noticed minimal breathlessness five days back that gradually increased to become severe, and she reported to the emergency department. The patient had diabetes mellitus with systemic hypertension and was on medication for five years. She underwent a vaginal hysterectomy for third-degree uterocervical prolapse in 2006. Histopathology suggested chronic cervicitis, cystic endometrial hyperplasia, and unremarkable myometrium.

On investigation, she had normocytic mildly hypochromic anemia with a few microcytes and neutrophilic leukocytosis on the peripheral smear. She had a hemoglobin level of 10.5 gm/dl and leukocytosis with a shift to the left (total leukocyte count: 18400 with 85% granulocytes). The pleural fluid was straw-colored. Cytology examination suggested 2-3 leukocytes per high power field with 75% polymorphs. Furthermore, the culture was suggestive of streptococcus pneumonia, and the patient was started on antibiotics as per the sensitive report. A two-dimensional (2D) echogram was suggestive of anterior wall dyskinesia with a thin wall and an ejection fraction of 35%. Moreover, the pleural effusion was ruled out to be of cardiac origin as it was unilateral; the patient did not have other signs and symptoms of congestive cardiac failure. Repeat fluid cytology on intercostal drain tube insertion was suggestive of sheets of well-isolated reactive mesothelial cells along with lymphocytes, macrophages, a few neutrophils, and debris in the blood-mixed, thin proteinaceous background with no evidence of malignant cells.

**Figure 2:**
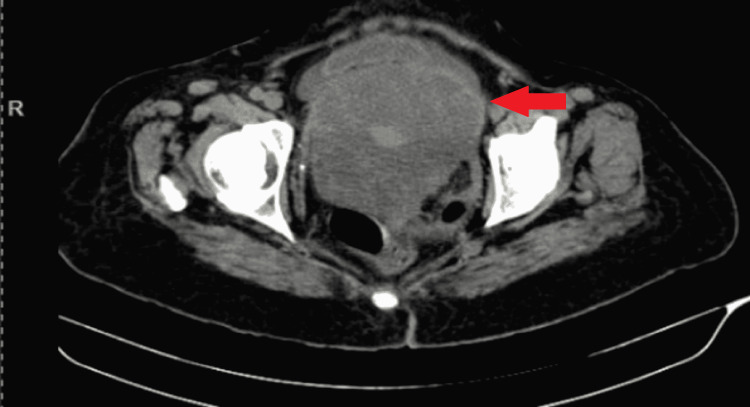
A contrast-enhanced CT scan revealed a large heterogeneously enhancing mass lesion in abdominopelvic region CT: Computed tomography

A contrast-enhanced CT scan of the abdomen revealed a large heterogeneously enhancing mass lesion with signs of neovascularization (Figure 2). The mass was arising from the pelvis and extending into the abdomen just above the umbilicus, measuring 20x20x17 cm in size. The lesion displaces the bilateral common iliac artery, external iliac artery, and small bowel loops and compresses the bladder, sigmoid, and rectum without signs of obstruction. CA 125 of the patient was 14 U/ml, falling in the normal range below 35 U/ml.

**Figure 3:**
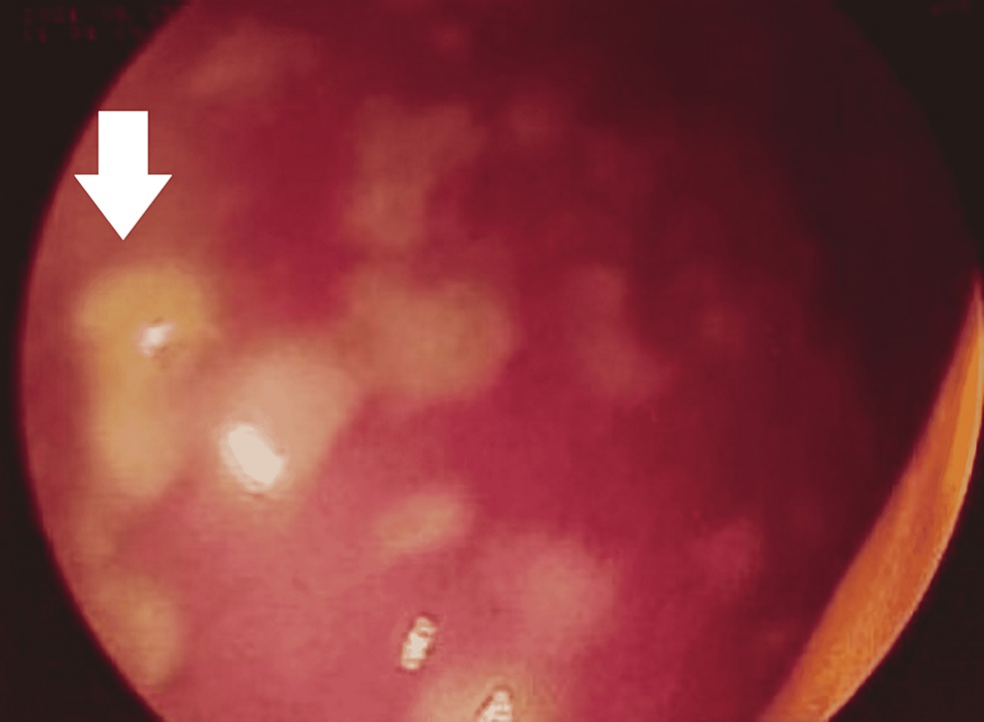
Colonoscopy image showing multiple whitish-yellow patches over the mucosa

The patient underwent colonoscopy for recurrent loose motions associated with abdominal cramps and occasional history of fever. She had also received medications multiple times in the last six months, including ciprofloxacin, metronidazole, pantoprazole, and pain relief. Extrinsic compression was pointed out in the sigmoid colon. Nevertheless, the scope could be negotiated proximally with minimal resistance. Colonoscopy showed inflamed colonic mucosa studded with multiple whitish-yellow patches with underlying small erosions covered with a pseudo-membrane over the rectum, sigmoid, and descending colon (Figure 3). Multiple biopsies were taken from varied sites, and histopathology examination showed sloughed epithelium with lymphoid aggregate and fibrin deposit. In addition to this, it had glandular crypts dilated by mucin and cellular aggregates forming the stems of the pseudo-membrane. All these clinicopathological features were consistent with the diagnosis of pseudomembranous colitis. However, this finding was unrelated to Meigs syndrome.

**Figure 4:**
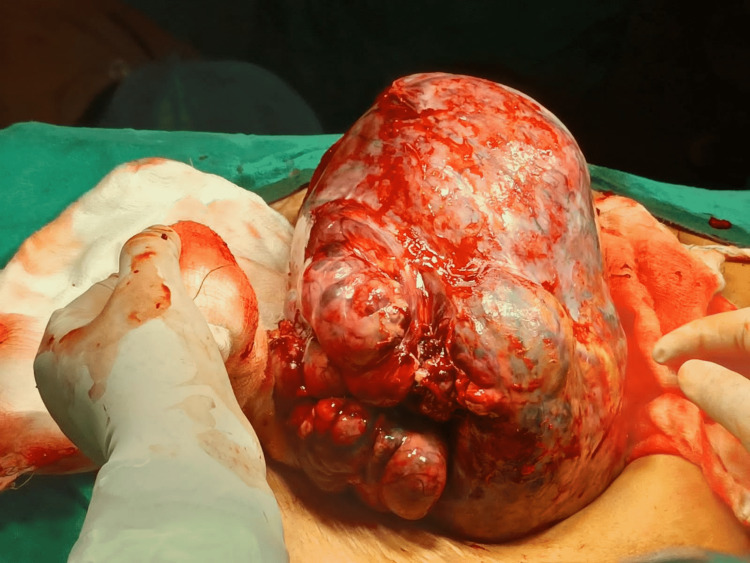
Intra-operative image of right ovarian mass

The patient was planned for excision of the mass and frozen section. Intraoperative, a large right ovarian mass lesion was identified (Figure 4). The bowel, bladder, and surrounding structures were not infiltrated. The ovarian mass was excised and sent for the frozen section. The left ovary was small and atrophic. However, it had nodular consistency and so was excised. The frozen section reported malignant granulosa theca cell tumor with omental infiltration by malignant ovarian cells, so lymph node dissection was performed. In the postoperative period, the malignant granulosa theca cell tumor diagnosis was confirmed on histopathology involving both ovaries. Postoperatively, the patient improved well and the pleural fluid drainage gradually reduced in quantity; the intercoastal drain was removed on the fifth postoperative. However, the patient continued to have medical management and was discharged on the tenth postoperative day.

## Discussion

Demons-Meigs syndrome is present in 1% of individuals with ovarian tumors [1,2]. The most common presentation is between the fifth and seventh decade of life with 67 to 70% of individuals being postmenopausal, except for a few patients who may present at a much younger age [27,28]. However, it is rare in the pubertal age group [27,28]. Fibromas are present in 1-3% of the ovarian mass [2,27]. Approximately 10-15% of all fibromas develop ascites and 1% develop hydrothorax, leading to Meigs syndrome [1]. The pleural effusion is more common on the right side (70%) and primarily exudates (88%), while left-sided and bilateral is 15% each [20].

The pathophysiology of the syndrome is not well understood. However, conjectural theories have been suggested. For example, Samanth and Black indicate that the ascites are formed due to the secretions of the tumor [29]. On the contrary, Meigs suggested the theory of peritoneal irritation leading to ascites by a larger ovarian tumor. Nevertheless, there is consensus that larger tumors more than 10 cm in dimension lead to ascites [29-31].

The pathophysiology of pleural effusion is documented by Meigs JV, Armstrong SH, and Hamilton HH by identifying ink in the pleural cavity after injecting it into the peritoneal cavity [32]. The same was reconfirmed by other authors [33,34]. Similarly, Vyas K, Krishnamurthy GT performed radionucleotide imaging to confirm trans-diaphragmatic spread. Furthermore, the occurrence of effusion commonly on the right side supports the theory of transdiaphragmatic migration of fluid. However, atypical Demons-Meigs syndrome with only pleural effusion without ascites cannot be explained by the transdiaphragmatic migration theory. And so, the theory of hormonal and vascular endothelial growth factor influence comes into the light [35,36]. It is proposed that the tumor secretes vascular endothelial growth factor, increasing the capillary permeability and leading to effusion. However, the question of its common occurrence on the right side cannot be explained by the hormonal mechanism. Furthermore, elevated CA 125 levels can be due to ovarian malignancy. In addition, CA 125 level may rise in benign conditions due to the inflammation of the omentum and the mesovarium [37]. There is no correlation between the dimensions of the ovarian mass and CA 125 values [38].

In systemic lupus erythematosus, ascites and pleural effusion are due to multiple factors like inflammation, lymph aggregation of plasma cells, vasculitis, and mediators of inflammation like fibroblast growth factor, cytokines, and vascular endothelial growth factor [39,40]. An individual with systemic lupus erythematosus and Demons-Meigs syndrome benefits from an anti-CD20 (cluster of differentiation 20) agent that reduces ascites and pleural effusion [41-43].

## Conclusions

Demons-Meigs syndrome is a rare clinical entity presenting benign ovarian mass, ascites, and hydrothorax. In the absence of ascites, this syndrome is labeled atypical. In addition, it is called pseudo-Meigs syndrome if a neoplastic ovarian mass is present instead of benign. Therefore, this is a case of atypical pseudo-Demons-Meigs syndrome as it has two unusual findings - a neoplastic tumor and an absence of ascites. The pathophysiology of Demons-Meigs syndrome is not well known. This patient has pleural effusion in the absence of ascites so it invalidates the theory of fluid transmigration and favors the hormonal and vascular endothelial growth factors theory. This case is particularly interesting as the patient had ischemic heart disease, systemic hypertension, and pseudomembranous colitis. The diagnosis is of utmost importance in such cases to avoid errors in treatment. Ascites and hydrothorax keep recurring in Demons-Meigs syndrome until the mass is excised, and it is crucial to excise the mass for the resolution of pleural effusion. Our case demonstrates that clinical knowledge of the syndrome is paramount in diagnosing and managing the syndrome. Furthermore, excision of the mass is the mainstay treatment for Demons-Meigs syndrome and its subtypes. Raised CA 125 levels in Demons-Meigs syndrome could be due to ovarian malignancy. However, it can also be elevated in benign conditions due to peritoneal irritation secretion, inflammation, or other causes. The clinical practice point to be considered is that thoracocentesis is a temporary solution in ovarian mass with pleural effusion. Excision of the mass should be done as soon as the patient is stabilized. In the treatment of ovarian mass, two schools of thought are suggested. The first is to do a pre-operative fine needle aspiration cytology (FNAC) followed by surgery. However, a more favored approach suggested by the author is excision and intra-operative frozen section as the treatment of choice in Demons-Meigs syndrome. This will not delay the treatment process and will help avoid multiple interventions for pleural effusion and ascites.
